# Camphorein

**Published:** 1874-02

**Authors:** 


Camphorein.—During the recent cholera epidemic in Vienna a new remedy, called *camphorein,* was used with great success in the hospitals. It is prepared simply by passing chlorine gas into pure turpentine oil until saturated. It gives a thick, heavy, oily fluid of brown color, with a strong smell of chlorine. It must be freed from muriatic acid, which may be done by washing with water. The remedy is applied by placing a portion in a flat vessel, and holding it to the patient to inhale. This indicates that oil of turpentine is the best absorbent of chlorine gas, and therefore can be employed with advantage in operations and other cases where chlorine is evaporated in large quantities.

